# Mechanisms of TLR4-Mediated Autophagy and Nitroxidative Stress

**DOI:** 10.3389/fcimb.2021.766590

**Published:** 2021-10-22

**Authors:** Kunli Zhang, Qiuyan Huang, Shoulong Deng, Yecheng Yang, Jianhao Li, Sutian Wang

**Affiliations:** ^1^ State Key Laboratory of Livestock and Poultry Breeding, Guangdong Key Laboratory of Animal Breeding and Nutrition, Institute of Animal Science, Guangdong Academy of Agricultural Sciences, Guangzhou, China; ^2^ Institute of Animal Health, Guangdong Academy of Agricultural Sciences, Guangzhou, China; ^3^ Institute of Laboratory Animal Sciences, Chinese Academy of Medical Sciences and Comparative Medicine Center, Peking Union Medical College, Beijing, China; ^4^ Guangdong Provincial Key Laboratory of Animal Molecular Design and Precise Breeding/Guangdong Provincial Research Center of Gene Editing Engineering Technology, Foshan University, Foshan, China

**Keywords:** TLR4, autophagy, nitroxidative stress, interaction, homeostasis

## Abstract

Pathogenic infections have badly affected public health and the development of the breeding industry. Billions of dollars are spent every year fighting against these pathogens. The immune cells of a host produce reactive oxygen species and reactive nitrogen species which promote the clearance of these microbes. In addition, autophagy, which is considered an effective method to promote the destruction of pathogens, is involved in pathological processes. As research continues, the interplay between autophagy and nitroxidative stress has become apparent. Autophagy is always intertwined with nitroxidative stress. Autophagy regulates nitroxidative stress to maintain homeostasis within an appropriate range. Intracellular oxidation, in turn, is a strong inducer of autophagy. Toll-like receptor 4 (TLR4) is a pattern recognition receptor mainly involved in the regulation of inflammation during infectious diseases. Several studies have suggested that TLR4 is also a key regulator of autophagy and nitroxidative stress. In this review, we describe the role of TLR4 in autophagy and oxidation, and focus on its function in influencing autophagy-nitroxidative stress interactions.

## Introduction

Autophagy is a physiological metabolic compensatory process of eukaryotic cells that maintains homeostasis. However, autophagy is also a conserved defense mechanism that evolved during cell evolution and which has an important role in the process of pathogenic infection. Generally, autophagy degrades or digests intracellular aging or damaged organelles, protein, metabolin, and even pathogenic microorganisms by autophagosome encapsulation and lysosomal binding (Nakatogawa et al., 2012). Autophagy of immune cells resists the invasion of pathogens by regulating cellular functions. For example, promoting autophagy helps to clear *Streptococcus*, *Helicobacter. pylori*, and *Pneumonia* (Wang et al., 2009; Ye et al., 2015; Nozawa et al., 2017). However, some microorganisms have evolved the ability to inhibit, escape, and even use autophagy, allowing them to continue to survive in the host body (Starr et al., 2012; Sharma et al., 2021). Moreover, autophagy is involved in the regulation of inflammation. Moderate autophagy maintains homeostasis by negatively regulating inflammation (Liu et al., 2016b; Zhong et al., 2016). Incorrect autophagy can damage the body and even cause organ failure by triggering cytokine storms (Lu et al., 2019; Zhao et al., 2019). How to regulate and use autophagy to protect the body is a hot spot of current research.

Nitroxidative stress is a state of physiological imbalance mainly related to the excessive production of reactive oxygen species (ROS) and reactive nitrogen species (RNS). It is well-known that nitroxidative stress is a general factor in many pathological conditions, including autoimmune diseases, infectious diseases, and tumor (Su et al., 2017; Smallwood et al., 2018). ROS and RNS also have dual-roles in the processes of removing pathogenic microorganisms and maintaining homeostasis. Low concentrations of ROS/RNS help maintain normal physiological metabolism and protect against infectious pathogens (Valko et al., 2007). However, the excessive ROS/RNS leads to an imbalance of redox states, metabolic disorders, and even damage of tissues and organs (Islam, 2017; Di Meo and Venditti, 2020). In addition, a serious pathogenic infection can induce severe nitroxidative stress followed by cytokine storm, resulting in the acute injury of tissues and organs (Mrityunjaya et al., 2020). Therefore, understanding how the body regulates nitroxidative stress levels is important.

The recognition of pathogenic microorganisms by pattern recognition receptors (PRRs) is the initial step in host innate immune responses. The Toll like receptor (TLR) family is an important class of PRRs that recognize a variety of bacteria and viruses, and induces the secretion of inflammatory cytokines and chemokines. Moreover, the activation of TLRs directly enhances the phagocytosis and killing capacity of innate immune cells that promotes the elimination of pathogens (Vasselon and Detmers, 2002). TLR4 is mainly expressed on the membranes of macrophages, dendritic cells and neutrophils. Activation of TLR4 is closely related to inflammation, autophagy, and nitroxidative stress during a pathogenic infection (Deng et al., 2020). In this review, we discuss recent studies on the activities of TLR4, autophagy, and nitroxidative stress during pathogenic microorganism infections, with a particular focus on the mechanisms of TLR4-mediated autophagy and nitroxidative stress.

## The Initiation of Autophagy and Nitroxidative Stress During Bacterial Infection

### Initiation of Autophagy

Autophagy is also known as type II programmed cell death. Moderate autophagy can degrade damaged organelles, denaturated macromolecules and intracellular pathogens, and then provide raw materials for cell metabolism (Levine and Kroemer, 2019; Zhu et al., 2019). Excessive autophagy and insufficient autophagy lead to disease (Lavandero et al., 2015). It was confirmed that autophagy is closely associated with proliferation of pathogenic microorganisms (Wang et al., 2020b). The stage and site of pathogenic infection, type of infected cell, and physiological state of the host body all affect the activity and outcome of autophagy. Therefore, identifying the initiation process of autophagy can help us understand its multiple functions.

Mammalian target of rapamycin (mTOR) is a key factor of autophagy initiation. It is widely known that mTOR is composed of two protein complexes mTORC1 and mTORC2, which have different structures and functions and are involved in many physiological processes. Unc-51-like kinase 1 (ULK1) is an indispensable component in autophagy vesicles, which can form the ULK1 complex with autophagy related 13 (Atg13), 200-kDa FAK family kinase interaction protein (FIP200), and Atg101 to induce autophagy (Chen et al., 2014; Hurley and Young, 2017). Under normal circumstances, mTORC1 suppresses autophagy by directly inhibiting the ULK1 complex activity. However, under stress conditions, mTORC1 is phosphorylated leading to the rapid disinhibition of ULK1 and Atg13, which triggers autophagy. In addition, mTORC1 also negatively regulates autophagy by phosphorylating autophagy/Beclin-1 regulator 1 (Ambra1) at Ser52 and 4’,6-diamidino-2-phenylindole (DAP1) at Ser3 and Ser51 (Koren et al., 2010; Cianfanelli and Cecconi, 2015). Conversely, mTORC2 indirectly inhibits autophagy through activation of the AKT/mTORC1 signaling axis. The mechanism involves the AKT-dependent phosphorylation inhibition of tuberous sclerosis complex 1/2 (TSC1/2) which induces Rheb activity that promotes mTORC1 activation (Bernard et al., 2020). Another major mTOR related signaling pathway is the AMP-activated protein kinase (AMPK) -mTOR pathway. The regulation of AMPK is extremely complex. Activation of AMPK phosphorylates TSC2 at Ser792 of mTORC1. Furthermore, PIM2 directly phosphorylates TSC2 at Ser1798 to activate mTORC1 (Lee et al., 2010; Lu et al., 2013). Moreover, AMPK initiates autophagy by directly phosphorylating ULK1 at Ser317 and Ser777 in response to stress (Pagano et al., 2014). In addition, a novel signaling axis AMPK- E3 ligase S-phase kinase-associated protein 2 (SKP2)-co-activator-associated arginine methyltransferase 1 (CARM1), was found to regulate autophagy activation by nutrient starvation (Shin et al., 2016). A recent study indicated that PKCα phosphorylated ULK1 at Ser423, which prevented autosomal formation and inhibited autophagy (Wang et al., 2018a). Atg13, ULK1, and TFEB can be negatively regulated by mTORC1. The secretion of TFEB during stress helped regulate the expression of genes involved in lysosomal biogenesis and lipid catabolism (Settembre et al., 2013).

The processes of autophagy initiation induced by different pathogenic microorganisms are also different. It was shown that *Salmonella typhimurium* invasion breaks through vesicles and enters the cytoplasm to selectively activate mTOR and degrade AMPK to escape autophagy (Liu et al., 2018). Streptococcus pneumoniae PavA activates the AMPK signaling pathway to induce autophagy in alveolar epithelial cells by inhibiting the mTOR pathway (Kim et al., 2017). In *H. pylori* infected gastric epithelial cells, inactivated transforming growth factor-β (TGF-β)-activated kinase 1 (TAK1) inhibits AMPK phosphorylation, as well as autophagy and cell survival (Lv et al., 2014).

### Formation of Nitroxidative Stress

Nitroxidative stress is a state of physiological imbalance mainly caused by excessive ROS and RNS. Under normal conditions, low levels of ROS/RNS trigger the hosts protective immune response, which is of great significance for anti-infection, anti-inflammatory, and tumor inhibition. However, excessive ROS/RNS directly leads to membrane rupture, DNA peroxidation damage, and cell dysfunction, such as loss of energy metabolism, changes in cell signal transduction, and gene mutation. During some pathogenic microorganisms infection, the host’s antioxidant system and metabolic balance of free radicals becomes disordered, resulting in nitroxidative stress.

ROS components include O_2_, H_2_O_2_ and –OH, and RNS components includes NO, NO_2_, and ONOO− (Stamler et al., 1992). ROS functions as a signaling molecule in a variety of intracellular processes, which lead to cell proliferation, apoptosis, and defense against microorganisms (Fratelli et al., 2005; Scherz-Shouval et al., 2007). Endogenous ROS is mainly induced by the mitochondrial respiratory chain (Zou et al., 2017). Specific enzymes of the NADPH oxidase (NOX) family and double oxidase family are involved in ROS production. These enzymes catalyze the conversion of intracellular O_2_ to O_2_^-^, and then O_2_^-^ is converted to H_2_O_2_ in the presence of superoxide dismutase. Subsequently, H_2_O_2_ reacts with metal ions to form –OH (Si et al., 2015). For example, sodion, a second messenger, interacts with phospholipids and controls oxidase in mitochondria. Furthermore, sodion helps transfer electrons from the substrate to oxygen ions, and subsequently promotes ROS formation and causes oxidative stress (Hernansanz-Agustin et al., 2020). Studies reported that SoxRS and OxyR are the main redox response transcription factors involved in oxidative stress in bacteria. OxyR senses H_2_O_2_ and organic peroxide, and SoxRS regulates O_2_^−^-mediated oxidative stress. In addition, other stress factors including RpoS and PerR are also involved in regulating oxidative stress responses (Fei et al., 2020). ROS and RNS are produced through different processes including ultraviolet irradiation, metal-catalyzed reactions, electron transport reactions, and inflammation (Valko et al., 2006). Many studies have shown that a variety of pathogens induce nitroxidative stress (Li et al., 2019). The increase of ROS/RNS production was found during microbial infection, or infection with hepatitis C virus, Herpes simplex virus type 1, *E. coli*, and *Salmonella* (Molteni et al., 2014; Rhen, 2019). When pathogenic microorganisms infect a host, ROS and RNS can be beneficial and detrimental *via* anti-inflammatory, anti-pathogen, protective immunity, or cytotoxicity functions (Umezawa et al., 1997). Moderate insulin-like growth factor 1 promoted mitochondrial ROS synthesis and NO synthase gene expression which participated in the elimination of *P. falciparum* (Drexler et al., 2014). Activation of TLR4 helped clear invading pathogens through the production of NO which was induced by increasing the activity of iNOS *via* guanosine triphosphate cyclohydrolase (Deng et al., 2015). Inhibition of mitochondrial ROS production impaired the ability of immune cells to kill *Salmonella typhimurium* in mice (West et al., 2011). In addition, excessive ROS/RNS was closely associated with tissue and organ damage (Deng et al., 2012). For example, excessive ROS triggered apoptosis by inducing the expression of NLRP3, caspase-1, and ASC (Dai et al., 2019).

### Antioxidant Systems

Because persistent and excessive nitroxidative stress can lead to host tissue damage, organisms have an antioxidant system that maintains redox homeostasis. There are two types of antioxidant systems: antioxidant enzymes and non-enzymatic antioxidants (Halliwell, 1996). Antioxidants help to mediate peroxidation by catalyzing the formation of active oxygen intermediates which lead to remove free radicals of oxygen and nitrogen. For example, the massive accumulation of ROS promotes the production of superoxide dismutase (SOD), which catalyzes the conversion of superoxide into O_2_ and H_2_O_2_ (Lipinski et al., 2010). Then, catalase scavenges peroxide by catalyzing the conversion of H_2_O_2_ into O_2_ and H_2_O (He et al., 2017). In addition, the GPx family and Trx system, endogenous antioxidants, also participate in the elimination of nitroxidative stress (Papp et al., 2007; Liu et al., 2014a). Moreover, several signaling pathways, of which nuclear factor erythroid 2-related factor 2 (NRF2) signaling is the most important, are involved in regulating nitroxidative stress. ROS directly oxidizes the cysteine residues on Kelch-like ECH-associated protein 1 (KEAP1) freeing NRF2 from the KEAP1-NRF2 complex. After free NRF2 translocates into the nucleus, it promotes the expression of multiple antioxidant genes by binding to their regulatory regions (Schieber and Chandel, 2014). Furthermore, NO^-^ triggers NRF2 transcription and the expression of ferroportin which activate the antioxidant system and nutrition immunity (Nairz et al., 2013). In turns, inhibiting NRF2 signaling increased ROS levels by suppressing the expression of HO-1 during *H. pylori* infection (Ko et al., 2016).

## Overview of TLR4: An Important Signal Transduction Protein

Studies over the past few decades have shown that the recognition of microbes is based on hosts genes encoding PRRs, which consist of TLRs, RIG-I-like receptors (RLRs), C-type lectin receptors (CLRs), and Nod-like receptors (NLRs) (Janeway, 2013). TLRs are Type I transmembrane proteins that recognize various microbial pathogen-associated molecular patterns (PAMPs). More than a dozen TLRs have been identified to date. Different TLRs can recognize different PAMPs in different cell compartments, including the cell membrane, endosomes, cytoplasm, and endosome (Akira et al., 2006). The correct localization of TLRs is crucial for the regulation of signal transduction. Furthermore, cell type specific signaling downstream of TLRs determines specific innate immune responses (Kawasaki and Kawai, 2014). Generally, TLR signaling is divided into two types: MyD88-dependent and the MyD88-independent signaling pathways. Both pathways activate downstream signaling molecules to promote the production of pro-inflammatory cytokines, chemokines, and type I interferon (IFN) which help remove pathogens (Yamashita et al., 2012). Here, we mainly focus on studies of TLR4 which is also involved in autophagy and nitroxidative stress.

### TLR4 Signal Transduction

TLR4 is known for recognizes a broad variety of substances including bacterial lipopolysaccharide (LPS), viral structural protein, fungal mannan, and parasitic glycoinositolphospholipids and has a key role in activating innate immunity (Kawai and Akira, 2011; Mu et al., 2011). For example, when Gram-negative bacteria infect a host, bacterial LPS is directly recognized by TLR4 in the presence of LPS binding protein, CD14, and myeloid differentiation factor 2 (MD-2). Subsequently, TLR4 undergoes oligomerization and recruits downstream adaptor proteins through its Toll-interleukin-1 receptor domains, which initiates signal transduction (Lu et al., 2008). Similar to other TLRs, TLR4 also has two types of signal transduction. After TLR4 is combined with MyD88, phosphorylated IRAK-4 activates the TRAF6-TAK1-NF-κB/MAPK signaling axis which promotes the release of inflammatory factors, such as IL-1β, IL-6, and TNF-α (Monlish et al., 2016; Dajon et al., 2017). Theses cytokines are responsible for inflammation, autophagy and nitroxidative stress. In the MyD88-independent pathways, TRAM is selectively recruited to TLR4 to link TLR4 and TRIF, which induces the production of type I IFN and other proinflammatory cytokines through the activation of IRF3, NF-κB, and MAPK signaling (Kawai and Akira, 2011).

### TLR4-Mediated Innate Immune Responses Have Dual Functions

However, when TLR4 is overactivated or its negative regulatory system is obstructed, TLR4 can induce endotoxic shock, autoimmune disease, and even cytokine storm where the immune system attacks the host. The SARS-CoV-2 virus has spread worldwide since 2020, infecting billions of people and killing millions of people (Harrison et al., 2020). Cytokine storm is thought to be an important cause of death in patients with severe and critical COVID-19, which is caused by the SARS-CoV-2 virus (Chen et al., 2020). Compared with a severe influenza group, PBMCs from COVID-19 patients showed a high inflammatory response, and a marked TNF/IL-1β driven inflammatory response. IFN, TNF-α, and IL-1β co-drive inflammatory responses in the monocytes of patients with severe COVID-19, but not in those of patients with mild COVID-19 (Lee et al., 2020). Further research showed that TLR4 is probably involved in recognizing the spike protein of SARS-CoV-2 and then inducing inflammatory responses (Bhattacharya et al., 2020; Choudhury and Mukherjee, 2020). After SARS-CoV-2 virus infects a host, the SARS-CoV-2 spike trimer directly binds to TLR4 and the trigger section of IL-1β. Inhibiting TLR4 completely blocks SARS-CoV-2-induced IL-1β. In contrast, ACE2-deficient or TMPRSS2-inhibition did not affect SARS-CoV-2-induced IL-1β (Zhao et al., 2021). This indicates that if TLR4 signals are not effectively controlled, they can seriously threaten health. Several tightly-regulated mechanisms regulate TLR4 signal transduction to avoid excessive immune response (**Table 1**) (Liu et al., 2016a).

**Table 1:** The negative regulatory molecules and targets of TLR4 signaling.

Regulated TLRs	Negative regulator molecule	Target	Refs
TLR4	RP105	Competing ligands for TLR4	(Liu et al., 2003)
TLR4/9	Rab7b	Promoting TLR4 degradation, inhibiting NF-κB/MAPK	(Wang et al., 2007)
TLR4	SHIP1	Inhibiting combination of TLR4 and MyD88	(Cekic et al., 2011)
TLR4/7/9	SOCS-1	Inhibiting IRAK activity	(Mansell et al., 2006)
TLR3/4	SARM/TRAF1/TRAF4	Inhibiting TRIF	(O’Neill and Bowie, 2007; Ermolaeva et al., 2008)
TLR4	Tollip/SHP1	Inhibiting self-phosphorylation of IRAK1	(O’Neill et al., 2003; Das et al., 2012)
TLR4	ATF3/Notch	Inhibiting NF-κB	(Kim et al., 2010; Yao et al., 2013)
TLR3/4	Rhbdd3/Ash1l	Inhibiting NEMO ubiquitination	(Xia et al., 2013; Liu et al., 2014b)
TLR4	IRG1/USF-1	Promoting A20 activity	(Shi et al., 2005; Tiruppathi et al., 2014)
TLR3/4/9	Zc3h12a	Promoting degradation of IL-6 and IL-12p40	(Chen et al., 2018)
TLR4	Tet2/Daxx	Promoting histone deacetylation, inhibiting IL-6	(Yao et al., 2014; Liang et al., 2020)
TLR2/4/5	miR-146a	Inhibiting IRAK and TRAF6 activity	(Chassin et al., 2010)
TLR4	miR-21	Inhibiting PDCD4	(Sheedy et al., 2010)
TLR2/3/4	Setdb2	InhibitingH3K9me3 levels in Cxcl1 promoter region	(Das et al., 2016)

## Regulatory Relationships Among TLR4, Autophagy, and Nitroxidative Stress

In previous studies, researchers mainly investigated the functions of TLR4 with regard to the activation of innate immune responses and production of proinflammatory cytokines during infection. Recently studies have suggested that TLR4 is involved in regulating autophagy and nitroxidative stress during various physiological states (Wang et al., 2016; Wang et al., 2018b; Wang et al., 2020a). Here, we summarize signaling pathways that connect TLR4, autophagy, and nitroxidative stress, and discuss their triangular relationships that affect cellular homeostasis.

### Key Signaling Nodes That Link TLR4, Autophagy and Nitroxidative Stress

#### mTOR Signaling Pathway

Identifying the signaling pathways shared by TLR4, autophagy and nitroxidative stress help us better understand the process of host resistance to pathogens. mTOR is involved in many cellular physiological processes, such as inflammation, energy metabolism, oxidation and autophagy (Zhou et al., 2018). The TLR4-MyD88-MAPK and TLR4/PI3K/Akt signaling pathways affect mTOR activity and influence autophagy (Huang et al., 2020). Generally, nitroxidative stress affects the phosphorylation of AMPK and PI3K signaling, which then influence autophagy (Hinchy et al., 2018; Mistry et al., 2019). Mechanistically, bacteria activate mTORC1 *via* the upstream TLR4-PI3K-Akt signaling cascade, which inhibits autophagy by suppressing autophagy initiating kinase ULK1 (Shariq et al., 2021). Down regulating TLR4 and MyD88 promoted autophagy by suppressing the phosphorylation of MAPK, mTOR and p65. However, activated TLR4-TRIF and TLR4-MAPK signaling pathways induced autophagy by promoting the dissociation of Beclin 1 and Bcl-2. The release of Beclin 1 from Bcl-2 biased cells toward autophagy (Shi and Kehrl, 2008). Then, mTOR-dependent autophagy precisely regulates downstream NF-κB activation, which leads to the production of nitroxidative stress (Zhou et al., 2018). ROS and RNS signaling were demonstrated to be related to TLR4-dependent NF-κB activation and inflammatory cytokines production (Weigert et al., 2018). The membrane-associated enzyme complex NADPH acts as a key intermediate in regulating the TLR4-mediated production of ROS. Furthermore, the inhibition of mTOR or mTORC1 down-regulated the TLR4-mediated production of ROS and RNS. mTOR-induced the expression of NOX2 and NOX4, which are essential for ROS formation (Sohrabi et al., 2018).

#### TRAF6 Signaling Pathway

**Figure 1:**
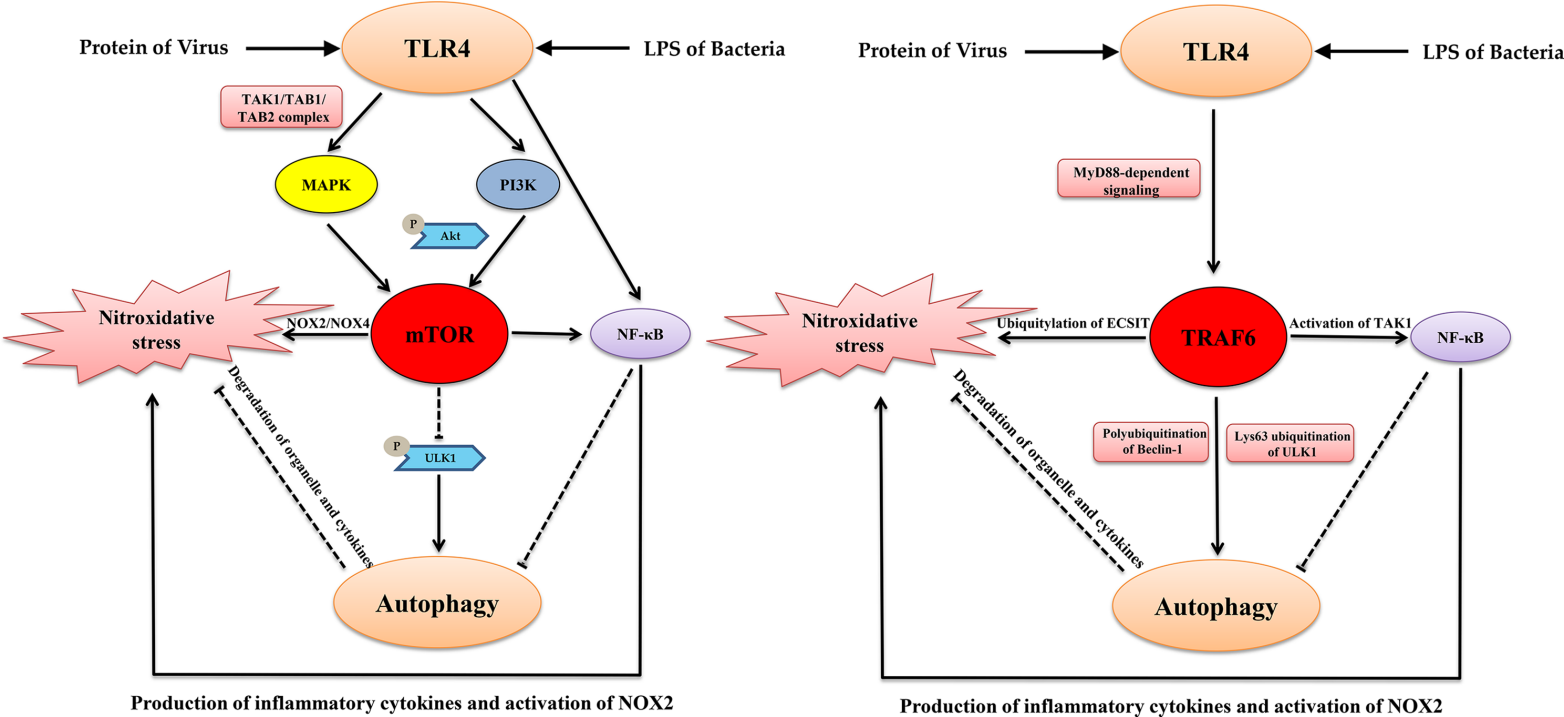
The key signaling nodes that link TLR4, autophagy and nitroxidative stress. TLR4, Autophagy, and Nitroxidative Stress: A Triangular Relationship That Affects Cellular Homeostasis.

TLR4-MyD88 signaling activates of TRAF6, another important factor that links autophagy and oxidation. TRAF6 promotes mtROS production during LPS stimulation or bacterial infection through the direct ubiquitylation of domains of evolutionarily conserved signaling intermediates in Toll pathways (ECSIT), which is a key mitochondrial respiratory chain assembly factor (West et al., 2011; Wi et al., 2014). Moreover, the inhibition of TRAF6 ubiquitin-ligase activity suppresses autophagy (Min et al., 2018). Mechanistically, activated TRAF6 promoted the K63-linked polyubiquitination of Beclin-1, which is essential for the initiation of autophagy (Lee et al., 2018). Furthermore, activated TRAF6 also induced the stabilization and activation of ULK1, which is the most important factor for the biogenesis of autophagosomes by inducing its Lys63 ubiquitination (Han et al., 2019). In addition, under non-autophagic conditions, mTOR induces the phosphorylation of Ambra1. Then, activated Ambra1 interacts with TRAF6 to mediate the ubiquitination of ULK1 that further inhibits the initiation of autophagy (Nazio et al., 2013). Furthermore, TRAF6 is necessary for the translocation of mTORC1 to lysosomes. The p62/TRAF6 complex induces the activation of mTORC1 *via* the K63-linked polyubiquitination of mTOR (Linares et al., 2013). These intriguing studies indicate that mTOR and TRAF6 are factors joints that connect TLR4 signaling, autophagy, and nitroxidative stress (**Figure 1**).

#### TLR4 and Nitroxidative Stress Induce Autophagy

**Figure 2:**
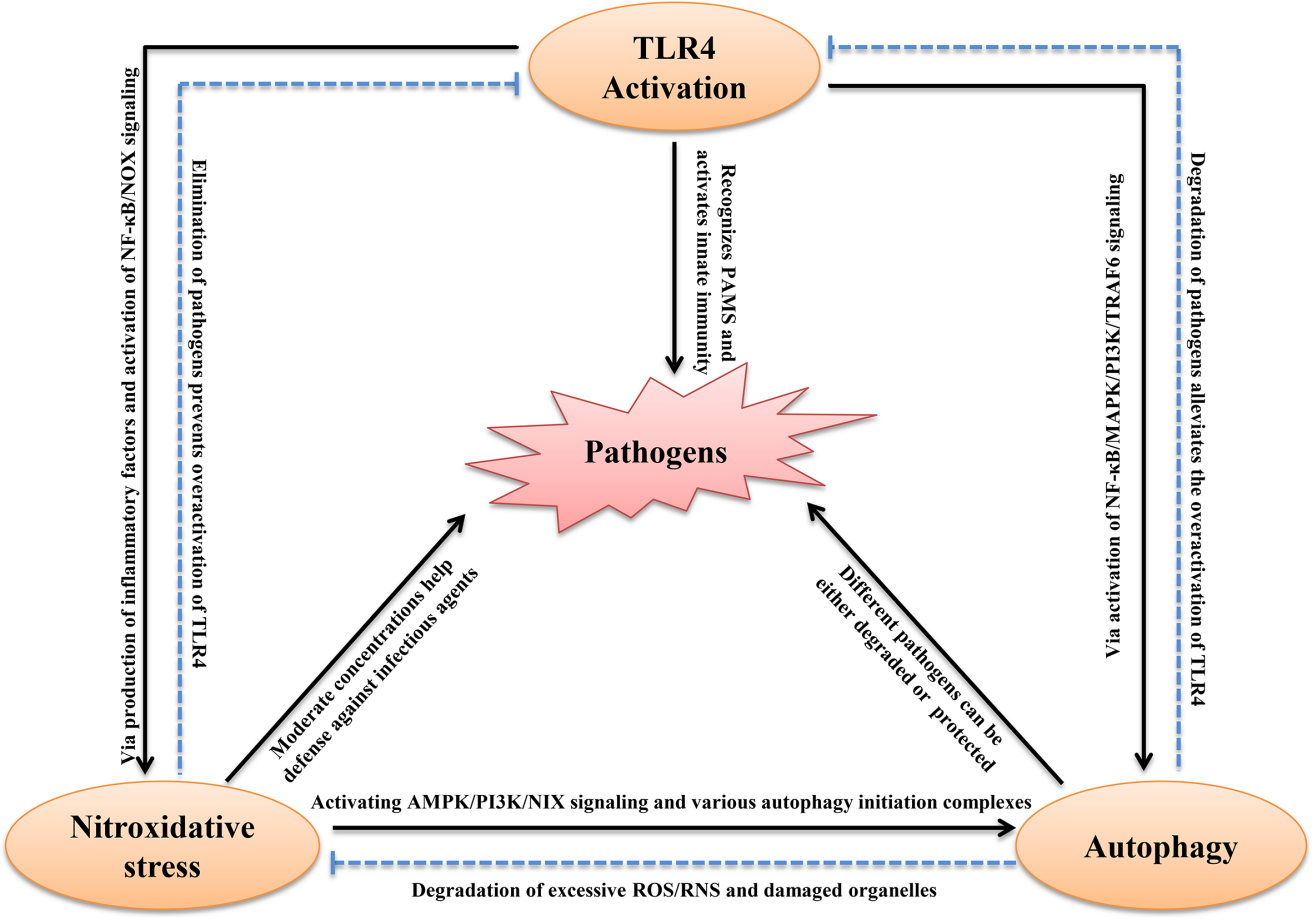
Schematic diagram of the interplay between TLR4, autophagy and nitroxidative stress in infectious Diseases. See text for explanation.

The process of TLR4 activation is always accompanied by autophagy and nitroxidative stress in host immune cells (**Figure 2**). For example, *mycobacterium tuberculosis* infection, recognized by TLR4, induced SIRT3-mediated autophagy and nitroxidative stress in mouse BMDM (Kim et al., 2019). *E. coli* LPS stimulation also induced autophagy and nitroxidative stress *via* MAPK signaling in macrophages (Wang et al., 2020a). The host recognition of bacteria is the first step for activation of the immune system. Generally, TLR4 recognizes PAMPs and induces the secretion of inflammatory cytokines, including type I IFN, which induces the production of ROS/RNS. Moderate intracellular oxidation is an important method for the host to eliminate pathogenic microorganisms. Evidence suggests that TLR4 interacts directly with NADPH oxidase. Indeed, the TIR-domain of TLR4 interacts directly with the carboxy-terminal region of Nox2/4 under LPS stimulation (Park et al., 2004). Activated NADPH oxidase induces the transmembrane transport of electrons and production of ROS. Furthermore, TLR4 promotes the expression of iNOS *via* NF-κB signaling. This promotes NO production that suppresses SOD activity and promotes MDA production, which aggravates nitroxidative stress (Li et al., 2019). In addition, TLR4-mediated pro-inflammatory cytokines, such as TNF-α and IL-1β, and also induces ROS (Sasaki et al., 2008). Moreover, inhibiting the expression of TLR4 significantly decreased the level of autophagy (Kandadi et al., 2012). The excessive accumulation of ROS/RNS is harmful to homeostasis and can cause body oxidative damage in the host. It is widely thought that ROS/RNS induces autophagy, which maintains oxidation intermediates at a low level. ROS directly phosphorylated the p65 subunit of NF-κB at Ser-536, which activated the autophagy receptor P62 (Song et al., 2017). In addition, many oxidation intermediates promote the transcription of *BNIP3* and *NIX*, which dissociate Beclin-1from Bcl2 to induce autophagy (Mahalingaiah and Singh, 2014; Xu et al., 2020). Moreover, H_2_O_2_ mediated the binding of AMPK and GSH which helped phosphorylate the ULK1 complex leading to autophagy (Filomeni et al., 2015). Furthermore, excessive RNS and ROS cause DNA damage (Wiseman and Halliwell, 1996). Upon DNA damage, Ataxia telangiectasia mutated promoted AMPK phosphorylation and the p53-dependent expression of autophagic genes, including ATG4, ULK1 and UVRAG (Hurley and Bunz, 2007; Fullgrabe et al., 2014). Nrf2 sensed cellular ROS or RNS, and then activated AMPK, which suppressed mTOR signaling leading to autophagy (Kapuy et al., 2018). In addition, the inhibition of ROS by *N*-acetyl-l-cysteine abolished autophagic flux in porcine trophectoderm cells and ROS production activated MAPK and PI3K/Akt pathways, which were involved in this process (Luo et al., 2019). As mentioned above, TLR4 regulated autophagy *via* the TLR4-MyD88-MAPK/NF-κB and TLR4/PI3K/Akt/mTOR signaling pathways. However, whether TLR4 activation has an active role in the induction of autophagy remains controversial. Most studies have shown that LPS or infection can induce autophagy *via* TLR4 signaling, and that the knock-down/knock-out of TLR4 down-regulates autophagy (Chen et al., 2015; Zhao et al., 2016; Wang et al., 2020a). However, a study reported that the activation of TLR4 by bacterial LPS inhibited autophagy through the MyD88-TAK1-MAPK-dependent phosphorylation of mTOR (Zhou et al., 2018). Another recent study showed that the intraperitoneal injection of LPS promoted neuroinflammation by activating TLR4, inhibiting autophagic markers, and inducing excessive oxidation intermediates (Jamali-Raeufy et al., 2021a). We hypothesize that this opposite result is related to the different concentrations and duration of stimulation or different types of cells used. However, there is no doubt that TLR4 is involved in regulating autophagy.

#### Autophagy Regulates Reactive Intermediates Production and TLR4 Activity

A recent study reported autophagy also regulated nitroxidative stress. Autophagy often helps remove damaged organelles and excess metabolic intermediates to maintain redox homeostasis in all types of cells (Kroemer et al., 2010). Autophagic dysfunction results in mitochondrial damage and the accumulation of oxidative intermediates, which promote the production of proinflammatory cytokines that can lead to ROS-mediated cell death (Larabi et al., 2020). Deletions of autophagy-related genes, such as *ATG5* and *ATG16* promoted high cellular ROS levels (Asano et al., 2017; Saxena et al., 2018). Moreover, nitroxidation intermediates have dual roles in the regulation of autophagy. NO activates autophagy *via* mTOR activity. Furthermore, NO accumulation triggered nitrosative stress, which promoted ATM/LKB1/AMPK/TSC2 signaling cascades that initiated autophagy *via* the inhibition of mTORC1 (Tripathi et al., 2013). NOS, another nitroxidation intermediate, interacted with PINK1 to activate selective autophagy by triggering the translocation of Parkin (Han et al., 2015). To date, no studies have shown that autophagy directly eliminates RNS. However, evidence has shown that autophagy removes substances that induce RNS production. For example, autophagy eliminated damaged mitochondria and inflammatory cytokines to reduce RNS accumulation (Shi et al., 2012; Kaminskyy and Zhivotovsky, 2014). Therefore, autophagy can be considered a non-canonical antioxidant system that helps to degrade excessive ROS/RNS. The intracellular redox state can affect the fate of cells, and the timely removal of oxidizing molecules is beneficial to the maintenance of homeostasis. Following the removal of pathogenic microorganisms by autophagy and nitroxidative stress, the activation of TLR4 is alleviated. During infection by pathogenic microorganisms, this physiological homeostasis helps to eliminate pathogens or helps pathogens survive. Conversely, if this homeostasis is disrupted, the host will be seriously challenged. The above studies have identified extensive crosstalk between TLR4, autophagy and nitroxidative stress (**Figure 2**).

## Concluding Remarks

As a pattern recognition receptor, TLR4 is involved in mediating pathogen-inducing inflammation. Moderate inflammatory responses help fight against pathogenic infections, but the excessive activation of inflammatory responses can trigger cytokine storm, which causes the immune system to attack the host leading to multiple organ failure and even death. Autophagy and nitroxidative stress are involved in the regulation of inflammation, and TLR4, autophagy, and nitroxidative stress have vital roles during pathogenic microorganism-induced host immune responses. Following the pathways of autophagy and nitroxidative stress and the signaling transduction of TLR4 has indicated a strong connection between them. Studying the detailed regulatory relationships between TLR4, autophagy and nitroxidative stress might help enhance our understanding of the interactions between pathogenic microorganisms and hosts. The activation of TLR4 always contributes to autophagy and the production of reactive intermediates including ROS/RNS. mTOR and TRAF6 are key factors that connect TLR4 signaling, autophagy and nitroxidative stress. Furthermore, promoting mTOR and TRAF6-dependent autophagy and nitroxidative stress *via* upstream TLR4-MyD88-NF-κB/MAPK and TLR4-TRAF6 signaling pathways, and downstream NOX, AMPK signaling and inflammatory cytokines regulates the elimination of pathogens and maintenance of body health. Furthermore, nitroxidative stress can induce autophagy through the activation of NF-κB, AMPK, and Nrf2 pathways. Then, autophagy helps to degrade excessive ROS/RNS to balance the intracellular redox state.

An important question is how to harness the links between TLR4, autophagy, and nitroxidative stress to control pathogenic microorganism infections. Many natural substances and small molecular compounds were reported to control autophagy and oxidation by regulating TLR4 activity (Jamali-Raeufy et al., 2021b). Of note, TLR4 activity, autophagy and oxidative stress all affect the survival of pathogenic microorganisms. According to the above ideas, we should identify targets from the signal pathways or key node proteins involved in TLR4/autophagy/nitroxidative stress simultaneously. Among them, mTOR, TRAF, NF-κB and their upstream and downstream molecules are worth investigating. In addition, we should choose different treatment strategies according to the types of pathogenic microorganisms and the characteristics of the infected organs. Finally, there are some outstanding questions: What are the differences between different TLR4 downstream signaling-mediated autophagy and nitroxidative stress pathways? Are TLR activation, autophagy and nitroxidative stress caused by infection and non-infectious diseases different? What are the specific molecular mechanisms and processes of the autophagic degradation of ROS/RNS? What are the detailed mechanisms of the activation of autophagy by endogenously produced RNS? Does excessive autophagy help to eliminate the ROS/RNS? If so, what is the relationship between excessive autophagy-mediated cell death and low ROS/RNS levels? These questions need to be answered in future studies.

## Author Contributions

SW, KZ, and SD conceived the manuscript. SW, KZ, and QH wrote the manuscript. SW, KZ, QH and YY prepared the Figure. SW and QH revised the manuscript. Funding acquisition was by SW and JL. All authors contributed to the article and approved the submitted version.

## Funding

The authors thank the following funding sources: National Natural Science Foundation of China (32002153, 32002298), Special fund for scientific innovation strategy-construction of high level Academy of Agriculture Science (R2019YJ-YB2004, R2019YJ-YB2005), Science and Technology Planning Project of Guangzhou (202102020177, 202102020385).

## Conflict of Interest

The authors declare that the research was conducted in the absence of any commercial or financial relationships that could be construed as a potential conflict of interest.

## Publisher’s Note

All claims expressed in this article are solely those of the authors and do not necessarily represent those of their affiliated organizations, or those of the publisher, the editors and the reviewers. Any product that may be evaluated in this article, or claim that may be made by its manufacturer, is not guaranteed or endorsed by the publisher.
